# Imbalances in the disposition of estrogen and naphthalene in breast cancer patients: a potential biomarker of breast cancer risk

**DOI:** 10.1038/s41598-020-68814-5

**Published:** 2020-07-16

**Authors:** Dar-Ren Chen, Wei-Chung Hsieh, Yi-Lun Liao, Kuo-Juei Lin, Yu-Fen Wang, Po-Hsiung Lin

**Affiliations:** 1 Comprehensive Breast Cancer Center, Changhua Christian Hospital, Changhua, 500 Taiwan; 2 Cancer Research Center, Department of Research, Changhua Christian Hospital, Changhua, 500 Taiwan; 3 Department of Laboratory Medicine, Da-Chien General Hospital, Miaoli, 360 Taiwan; 4 Department of Environmental Engineering, National Chung Hsing University, Taichung, 402 Taiwan; 5 Department of Surgery, E-Da Hospital, I-Shou University, Kaohsiung, 824 Taiwan

**Keywords:** Breast cancer, Tumour biomarkers, Breast cancer, Risk factors

## Abstract

Elevation of naphthoquinones and estrogen quinones, which are reactive metabolites of naphthalene and estrogen, is thought to be an important indicator of naphthalene- and estrogen-induced carcinogenesis. We compared background levels of naphthalene and estrogen quinone-derived adducts in serum albumin (Alb) from 143 women with breast cancer and 119 healthy controls. Cysteinyl adducts of naphthoquinones, including 1,2-naphthoquinone (1,2-NPQ) and 1,4-naphthoquinone (1,4-NPQ), and estrogen quinones, including estrogen-2,3-quinones (E_2_-2,3-Q) and estrogen-3,4-quinones (E_2_-3,4-Q), were characterized after adduct cleavage. Levels of estrogen quinones and naphthoquinones were positively correlated in healthy controls, but not in breast cancer patients (*p* < 0.05). Compared with controls, levels of 1,2-NPQ and E_2_-3,4-Q were elevated by two- to ten-fold in cancer patients (*p* < 0.001). To explore the correlation between estrogen- and naphthalene-derived quinone adducts and disease status, we performed linear discriminant analysis of the ratio of 1,2-NPQ-Alb to (1,2-NPQ-Alb plus 1,4-NPQ-Alb) versus the ratio of E_2_-3,4-Q-2-S-Alb to (E_2_-2,3-Q-4-S-Alb plus E_2_-3,4-Q-2-S-Alb) in patients and controls. These two groups were separable using albumin adducts of estrogen quinones and naphthoquinones, with 99.6% overall correct classification rate (overall accuracy). The findings of this study suggest that differences in the disposition of estrogen and naphthalene, and the subsequent elevation of cumulative E_2_-3,4-Q and 1,2-NPQ may serve as biomarkers of breast cancer risk.

## Introduction

Environmental factors and genetic predisposition are known to contribute to breast cancer risk^1–3^. Variation in the expression of estrogen bioactivation and deactivation genes may cause an imbalance in estrogen metabolism, resulting in elevated reactive quinone species and increased risk of breast cancer^1,2^. Increased serum estrogen and modulation of estrogen disposition are both associated with breast cancer development^4,5^. Mitogenesis driven by the estrogen receptor plays a critical role in estrogen carcinogenicity^6^. Conversion of 17β-estradiol (E_2_) to reactive metabolites, including 2-hydroxyestradiol (2-OH-E_2_) and 4-hydroxyestradiol (4-OH-E_2_), is mediated by CYP1A1/1A2 and CYP1B1^7–9^. Estrogen catechols undergo oxidation to form estrogen quinones, including estrogen-2,3-quinone (E_2_-2,3-Q) and estrogen-3,4-quinone (E_2_-3,4-Q)^10,11^. Accumulation of these estrogen quinones, particularly E_2_-3,4-Q, along with the subsequent generation of abasic sites and other pro-mutagenic DNA damage, further contribute to the initiation of estrogen-induced carcinogenesis^5,12,13^ and elevate the risk of developing breast cancer^14,15^.

Polycyclic aromatic hydrocarbons (PAHs) are ubiquitous environmental pollutants that form during incomplete combustion of organic material. Chronic exposure to PAHs is a risk factor for skin and lung cancer in humans^16^, and epidemiological studies have reported an association between PAHs and increased risk of breast cancer^17^. The carcinogenic properties of PAHs are primarily due to bioactivation of PAHs, which results in diol epoxides and the subsequent generation of DNA adducts^18,19^ and mutations^20,21^. Some PAHs are strong inducers of the aryl-hydrocarbon receptor (AhR) and capable of inducing transcriptional response in genes AhR-regulated genes, including genes responsible for biotransformation of estrogen to reactive quinonoid metabolites^16^. Naphthalene, a congeneric form of PAHs, causes a broad spectrum of toxicity in laboratory animals and humans^22,23^. Naphthalene is metabolized to form naphthalene epoxide cytochrome P450 1A1/1A2/2E1^24–27^. Epoxide hydrolase catalyzes the hydration of arene oxide intermediates to 1,2-dihydronaphthalene-1,2-diol 1,2-dihydro-1,2-naphthalenediol^24,28^. The oxidation of 1,2-dihydronaphthalene-1,2-diol to 1,2-naphthoquinone (1,2-NPQ) occurs via the intermediate metabolite 1,2-naphthalenediol. This oxidation is catalyzed by aldo–keto reductase enzymes, especially dihydrodiol dehydrogenase^29–31^. Alternatively, naphthalene epoxide may undergo spontaneous rearrangement to generate 1-naphthol and 2-naphthol. 1,2-NPQ can also be formed through the oxidation of 2-naphthol mediated by cytochrome P450 2E1/1A1/1A2^24^. Cytochrome P450 1A2 and 2D6 have been identified as the most active isoforms for the production of 1,4-naphthoquinone (1,4-NPQ) from 1-naphthol, which occurs via 1,4-naphthalenediol (NHQ)^24^. This compound is thought to initiate cancer via the activation and interaction of 1,2-NPQ with DNA to form depurinating adducts^32^. Albumin (Alb) adducts of quinonoid metabolites of naphthalene have been used as biomarkers of occupational and environmental exposure to PAHs, with increased naphthoquinone-derived Alb adduct levels detected in coke oven workers^33^. In a previous study, we used Alb adducts of quinonoid metabolites of naphthalene to estimate the body burden of naphthoquinones in human subjects in Taiwan^34^, concluding that relatively high naphthoquinones levels in serum may point to toxicological consequences. Additionally, we observed a positive correlation between levels of estrogen quinone- and naphthoquinone-derived protein adducts in serum derived from pregnant women^35^. However, the joint effects of imbalances in these adducts on breast cancer risk has not been reported.

To extend our previous research on estrogen quinone adducts in serum Alb on a broader scale, we examined the relationships between body burden of estrogen quinones with naphthoquinones in serum derived from breast cancer patients and controls and performed correlation analysis of levels of estrogen and naphthalene-derived quinone adducts with disease status. Our original protocol was refined to allow simultaneous analyses of estrogen quinone and naphthoquinone-derived adducts in serum Alb. For estrogen quinones, products of reactions between estrogen quinones and Alb are designated as E_2_-2,3-Q-1-S-Alb, E_2_-2,3-Q-4-S-Alb, and E_2_-3,4-Q-2-S-Alb, respectively, and those with naphthoquinones as 1,2-NPQ-S-Alb and 1,4-NPQ-S-Alb, respectively.

## Results

### Subjects’ characteristics

Mean age was 40 years (range 23–69) for controls (n = 119) and 51 (range 32–79) for breast cancer patients (n = 143). Mean body mass index was 22.7 (range 16.1–32.9) for controls and 24.7 (range 18.2–40.9) for breast cancer patients.

### Naphthoquinone-derived adducts in human serum Alb

**Figure 1:**
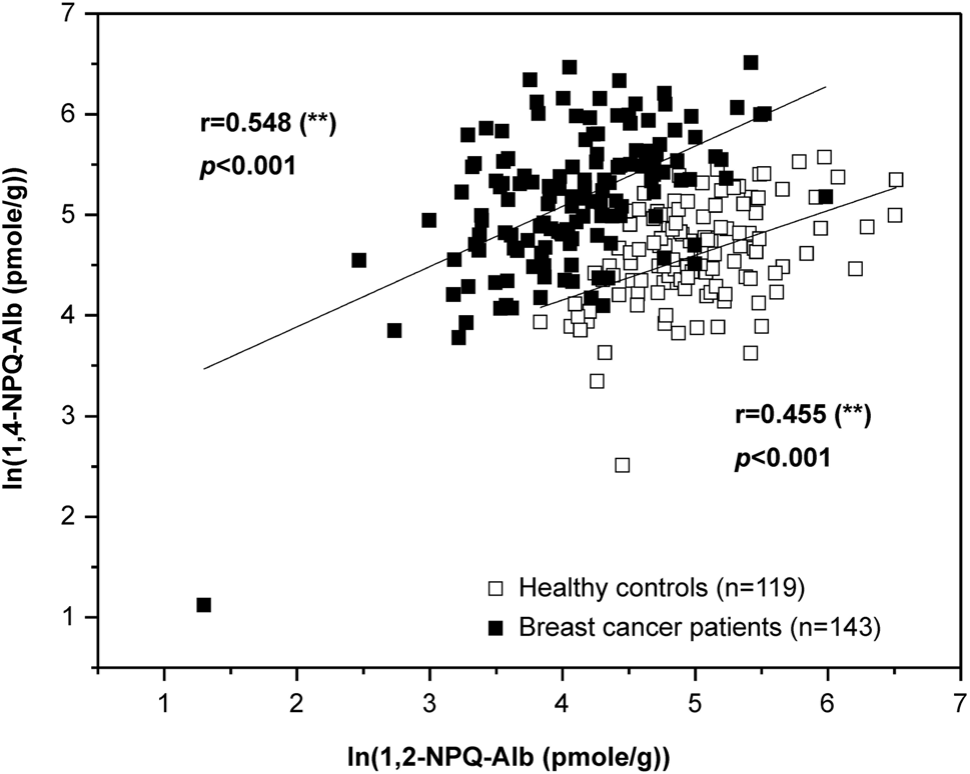
Linear regression of logged levels of 1,2-NPQ-Alb and 1,4-NPQ-Alb in breast cancer patients and controls. Filled square, breast cancer ptients; open square, healthy controls.

Cysteinyl adducts of both 1,2-NPQ and 1,4-NPQ were detected in serum Alb of all participants (Table 1). Median levels of 1,2-NPQ-Alb were 178 and 102 pmol/g in cancer patients (n = 143) and controls (n = 119), respectively, whereas median levels of 1,4-NPQ-Alb were 58.8 and 144 pmol/g, respectively. Levels of 1,2-NPQ-Alb were elevated two-fold in cancer patients compared to controls (*p* < 0.001). By contrast, levels of 1,4-NPQ-Alb were two times greater in controls than those in patients (*p* < 0.001). Ln (1,2-NPQ-Alb) correlated with ln (1,4-NPQ-Alb) in both breast cancer patients (r = 0.548, *p* < 0.001) and controls (r = 0.455, *p* < 0.001) (Fig. 1).

**Table 1:** Background levels of 1,2-NPQ- and 1,4-NPQ-derived Alb adducts in human serum derived from women with breast cancer and healthy controls.

Adducts	Patients (n = 143)	Healthy controls (n = 119)
*1,2-NPQ-Alb (pmole/g-Alb)*		
Mean (SD)	210 (130)^a^	113 (51.9)
Median	178	102
Range	ND^b^–674	28.3–263
*1,4-NPQ-Alb (pmole/g-Alb)*		
Mean (SD)	73.7 (52.3)	174 (108)^a^
Median	58.8	144
Range	ND^b^–397	46.2–674
Ratio of mean levels of 1,2-NPQ-Alb to 1,4-NPQ-Alb	2.85 (2.49)^b^	0.650 (0.480)

^a^, Statistical significance, p < 0.001. ^b^, Not detected. The limit of detection corresponds to 10 pmol/g for all adducts, assuming 10 mg protein was used for the assay.

### Estrogen quinone-derived adducts in human serum Alb derived from breast cancer patients and controls

**Figure 2:**
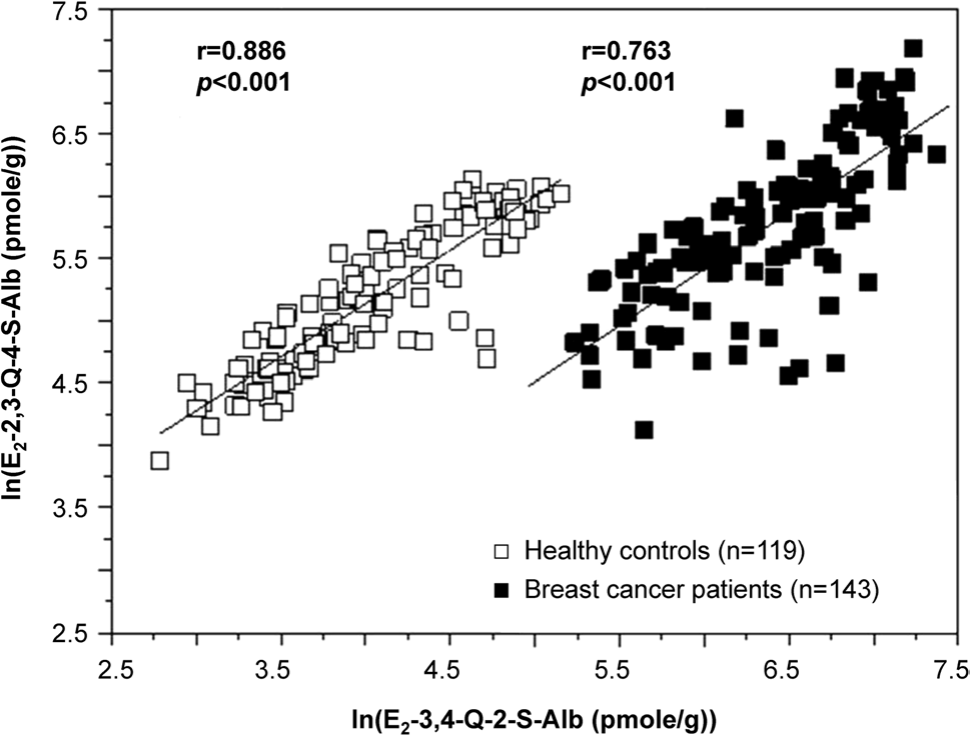
Linear regression of logged levels of E_2_-2,3-Q-4-S-Alb and E_2_-3,4-Q-2-S-Alb in breast cancer patients and controls. Filled square, breast cancer ptients; open square, healthy controls.

Cysteinyl adducts of E_2_-2,3-Q-4-S-Alb and E_2_-3,4-Q-2-S-Alb were also detected in serum Alb of all the participants (Table 2). The median levels of E_2_-2,3-Q-4-S-Alb in cancer patients (n = 143) and controls (n = 119) were 323 and 171 pmol/g, respectively, while E_2_-3,4-Q-2-S-Alb were 633 and 53.4 pmol/g. On average, levels of E_2_-2,3-Q-4-S-Alb and E_2_-3,4-Q-2-S-Alb were elevated two- and ten-fold in cancer patients compared to controls, respectively (*p* < 0.001). Ln (E_2_-2,3-Q-4-S-Alb) correlated with ln (E_2_-3,4-Q-2-S-Alb) in both breast cancer patients (r = 0.763, *p* < 0.001) and controls (r = 0.886, *p* < 0.001) (Fig. 2).

**Table 2:** Background levels of E_2_-2,3-Q-1-S-Alb and E_2_-2,3-Q-4-S-Alb, and E_2_-3,4-Q-2-S-Alb in serum derived from women with breast cancer and healthy controls.

Adducts	Patients^a^ (n = 143)	Healthy controls (n = 119)
*E*_*2*_*-2,3-Q-1-S-Alb (pmole/g-Alb)*		
Mean (SD)	ND^b^	ND^b^
Median	ND^b^	ND^b^
Range	ND^b^	ND^b^
*E*_*2*_*-2,3-Q-4-S-Alb (pmole/g-Alb)*		
Mean (SD)	400 (258)^c^	204 (111)
Median	323	171
Range	61.7–1,325	48–461
*E*_*2*_*-3,4-Q-2-S-Alb (pmole/g-Alb)*		
Mean (SD)	676 (338)^c^	66.8 (38.8)
Median	633	53.4
Range	188–1594	16.2–174
Ratio of mean levels of E_2_-3,4-Q-2-S-Alb to E_2_-2,3-Q-4-S-Alb	1.69 (1.37)^c^	0.330 (0.350)

^a^, Most estrogen quinone adduct data were previously analyzed and published ^35^. These data are included in this table for comparison with healthy controls. ^b^, Not detected. The limit of detection corresponds to 10 pmol/g for all adducts, assuming 10 mg protein was used for the assay. ^c^, statistical significance, *p* < 0.001.

**Figure 3:**
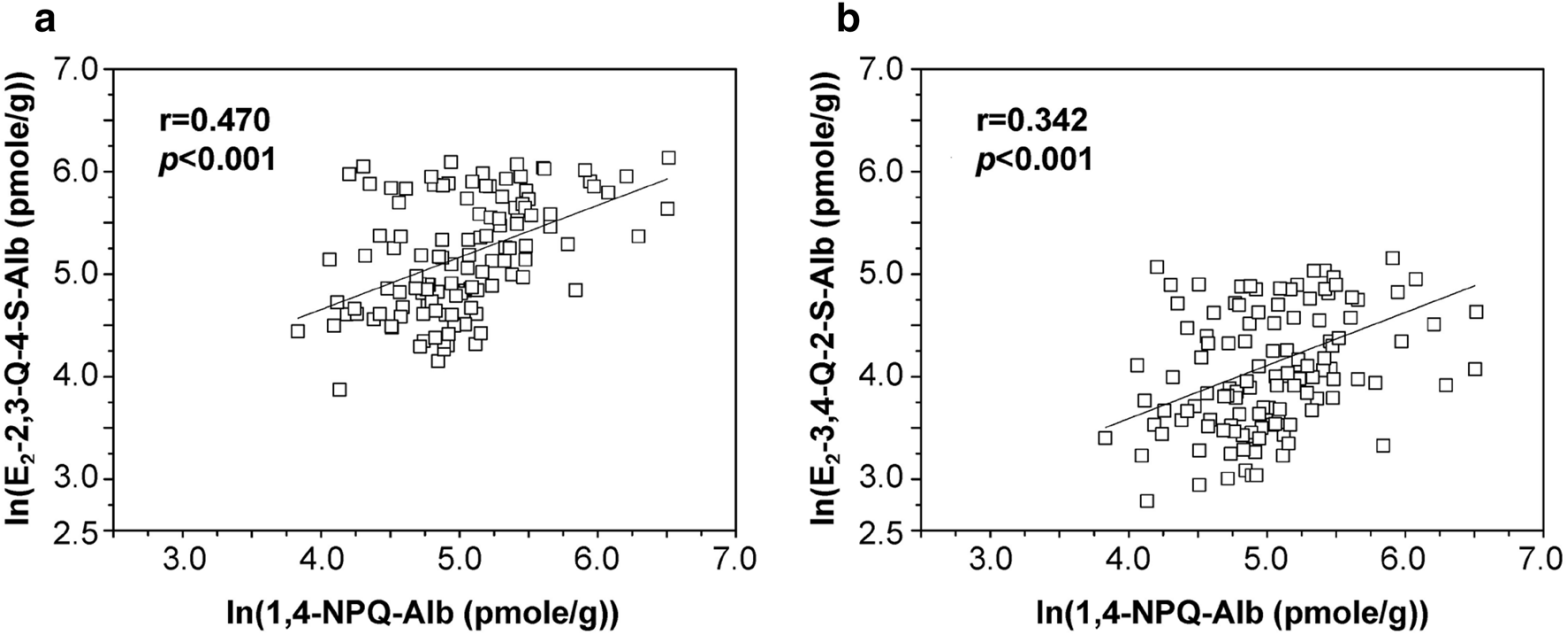
Linear regression of logged levels of 1,4-NPQ-Alb with (**a**) E_2_-2,3-Q-4-S-Alb and (**b**) E_2_-3,4-Q-2-S-Alb in healthy controls.

Correlation analysis between levels of estrogen quinone-derived adducts and naphthoquinone adducts was performed. Figure 3 depicts the linear regression of levels of ln (1,4-NPQ-Alb) with ln (E_2_-2,3-Q-4-S-Alb) and ln (E_2_-3,4-Q-2-S-Alb) and ln (1,2-NPQ-Alb) with ln (E_2_-2,3-Q-4-S-Alb) in healthy controls. The correlation coefficients (r) were estimated as 0.470 and 0.342 (*p* < 0.001) for E_2_-2,3-Q-4-S-Alb and E_2_-3,4-Q-2-S-Alb, respectively. Similarly, 1,2-NPQ-Alb was found to positively correlate with E_2_-2,3-Q-4-S-Alb in healthy controls (r = 0.259, *p* < 0.01). By contrast, no statistically significant association was observed between estrogen quinone-derived adducts and naphthoquinone adducts in cancer patients.

**Figure 4:**
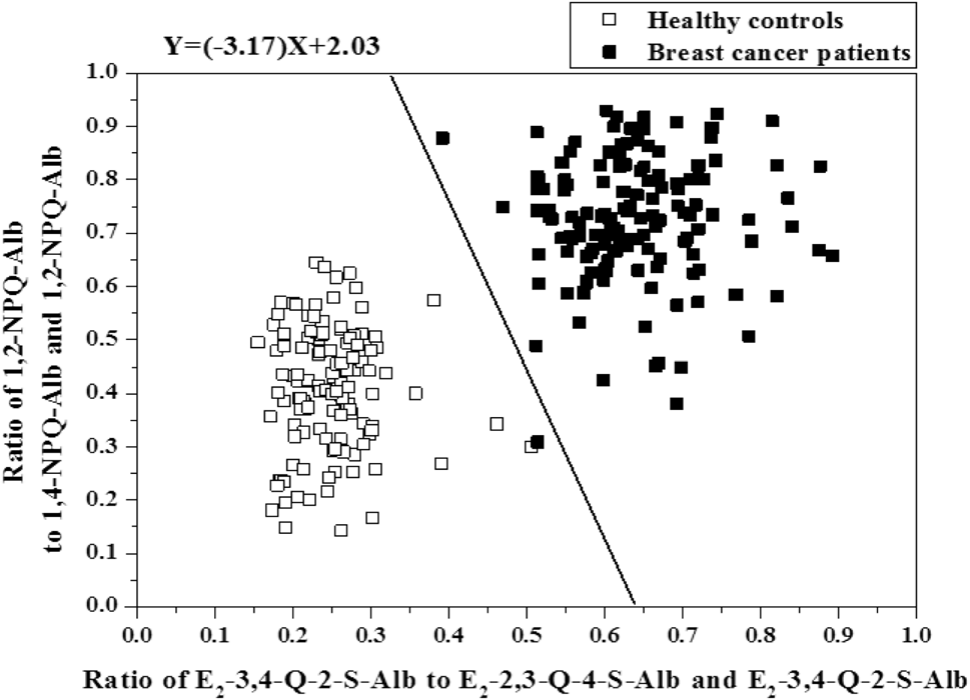
Ratio of 1,2-NPQ-Alb to 1,2-NPQ-Alb plus 1,4-NPQ-Alb verse ratio of E_2_-3,4-Q-2-S-Alb to E_2_-2,3-Q-4-S-Alb plus E_2_-3,4-Q-2-S-Alb in breast cancer patients and controls. Filled square, breast cancer ptients; open square, healthy controls.

To further explore the relationship between levels of estrogen and naphthalene-derived quinones protein adducts and disease status of breast cancer, we plotted the ratio of 1,2-NPQ-Alb to (1,2-NPQ-Alb plus 1,4-NPQ-Alb) versus the ratio of E_2_-3,4-Q-2-S-Alb to (E_2_-2,3-Q-4-S-Alb plus E_2_-3,4-Q-2-S-Alb) derived from breast cancer patients and controls (Fig. 4). The resulting combination was used as a linear classifier. The ratios of estrogen and naphthalene-derived quinone adduct classification are performed using linear discriminant analysis (LDA) classifier by calculating multivariate normal distribution density function for each class. Further investigation using LDA indicated that the two subject groups were separable using the Alb adducts of estrogen quinones and naphthoquinonesThe quantitative evaluation of LDA classifier shows that the number of the true positive and true negative subjects are 142 and 119, respectively. It achieves 99.6% overall correct classification rate (overall accuracy) for both classes in test dataset with 99.3% sensitivity and 100% specificity.

## Discussion

An imbalance of estrogen to quinonoid metabolites has been found to elevate the risk of developing breast cancer^12,36^. The underlying mechanism of this association may be the body burden of estrogen quinones and subsequent generation of DNA lesions, which ultimately increase mutation rate. The biotransformation of naphthalene to reactive quinonoid intermediates, including 1,2-naphthalenediol, NHQ, and their respective quinones, i.e., 1,2-NPQ and 1,4-NPQ, has been implicated in naphthalene-induced cytotoxicity and genotoxicity^37^. Naphthalene-derived quinonoids tend to induce reactive oxygen species formation and modification of intracellular redox status in human T47D cells, leading to further DNA damage and cell death^38^. There is evidence that 1,2-NPQ reacts with 2′-deoxyguanosine to form depurinating adducts and abasic sites^39^. If unrepaired, abasic sites can lead to mutations and cellular transformation. Our previous investigation of estrogen quinone- and naphthoquinone-derived protein adducts in serum Alb derived from healthy pregnant women indicated that cumulative body burden of 1,2-NPQ was associated with the extent of bioactivation of estrogen to reactive quinone species ^35^. This evidence may translate to an idea that imbalances in the disposition of estrogen and naphthalene to their specific quinone species plays a role in breast cancer risk. Direct investigation of the relation between body burden of reactive metabolites of estrogen and naphthalene quinones and breast cancer risk may enhance our understanding of the mechanistic pathways underlying this disease. As shown in Figs. 3, the findings in the correlation between levels of estrogen quinone-derived adducts and naphthoquinone adducts in healthy controls (except for 1,2-NPQ-Alb vs E_2_-3,4-Q-2-S-Alb ) but not in cancer patients reveal that factors besides PAH exposure modulate the bioactivation of estrogen to quinone species in cancer patients to a much greater extent when compared to controls.

Of note, the ratios of mean levels of 1,2-NPQ-Alb to 1,4-NPQ-Alb were estimated to be 2.85 and 0.650 for cancer patients and controls, respectively (Table 1). Similar observation was detected in estrogen quinone-derived adducts with ratios of mean levels of E_2_-3,4-Q-2-S-Alb to E_2_-2,3-Q-4-S-Alb of 1.69 and 0.330 for cancer patients and controls, respectively (Table 2). These patterns suggest that imbalance in the disposition of estrogen and naphthalene to their specific quinone species, i.e., E_2_-3,4-Q and 1,2-NPQ, is associated with risk of breast cancer. Levels of E_2_-3,4-Q and 1,2-NPQ were elevated two- to ten-fold in cancer patients compared to controls (*p* < 0.001). Further investigation using linear discriminant analysis demonstrated that the levels of Alb adducts of estrogen quinones and naphthoquinones could clearly distinguish breast cancer from healthy controls. These findings remain consistant as distinguished by age and body mass index (data not shown) and are compatible with the notion that the increase in breast cancer risk with increasing formation of specific quinone species is largely the result of the associated imbalance in metabolism^40^. Various environmental and genetic factors influence the production of estrogen and naphthalene quinones in humans^1,2,41^. It is possible that the association between cumulative burden of favorable quinone species, including E_2_-3,4-Q and 1,2-NPQ, and breast cancer risk could be partly explained by genotoxicity, which tends to generate depurinated adducts and abasic sites.

However, it is worth noting that this study might subject to the number of participants due to the limited resources. Further research is also warranted to investigate the relationship between breast cancer risk and polymorphisms in genes involved in disposition of estrogen and naphthalene.

In this study, we found that 1,2-NPQ-Alb levels in breast cancer patients were higher than in healthy controls, while 1,4-NPQ-Alb had an opposite trend. The levels of E_2_-2,3-Q-4-S-Alb and E_2_-3,4-Q-2-S-Alb were both higher in breast cancer patients than those of healthy controls. These findings provide further evidence to support the idea that the imbalance in the disposition of estrogen and naphthalene to their specific quinone species is associated with risk of breast cancer, especially E_2_-3,4-Q and 1,2-NPQ. By detecting the levels of Alb adducts of estrogen quinones and naphthoquinones, it is possible to clearly distinguish between breast cancer patients and healthy controls. Taken together, our findings support that differences in the disposition of estrogen and naphthalene, and the subsequent elevation of cumulative body burden of E_2_-3,4-Q and 1,2-NPQ, may serve as biomarkers of breast cancer risk.

## Methods

### Chemicals

In this study, organic solvents from TEDIA (Charlotte, NC, US), such as acetone, acetonitrile, ethyl acetate, and methyl alcohol, were used. Other chemicals used in this study were purchased from from Sigma-Aldrich Inc. (St. Louis, MO, US), unless otherwise stated. All the chemicals were used without further purification.

### Subjects

A hospital-based case control study was conducted to achieve the research goals. Subjects included women with a diagnosis of breast cancer and women with no evidence of breast disease, who were evaluated and/or treated at the medical center in central Taiwan. We randomly recruited 190 women with breast cancer (stage 0–4) and 205 controls between May 2009 and November 2012. Every effort was made to enroll cases prior to treatment and especially prior to surgery, hormonal therapy, radiotherapy, or chemotherapy, at the time of blood collection. The control group consisted of women coming to the hospital for a routine physical examination. All the participants provided sufficient venous blood for protein adduct analyses and completed questionnaires regarding age, body mass index, occupation, disease history, cigarette smoking, alcohol consumption, etc. Criteria for exclusion among cases and controls were: currently pregnant or lactating, currently taking antibiotics, smoking, or heavy alcohol consumption. Of those recruited, subjects with insufficient amount of albumin samples or with samples that were failed to complete analysis were also excluded. Ultimately, 143 breast cancer patients and 119 controls with no cancer history were enrolled in this study. The study protocol was approved by the Institutional Review Board of Changhua Christian Hospital, Taiwan (IRB no. 081219). All subjects’ written informed consent and all methods in this study were performed in accordance with the Declaration of Helsinki and relevant guidelines and regulations.

### Synthesis of isotopically-labeled protein-bound internal standards

Isotopically-labeled protein-bound internal standards were synthesized from [^2^H_5_]-E_2_ (C/D/N Isotope, Canada H9R 1H1) following the procedure by Butterworth^10^ with minor modifications^42^. Isotopically-labeled protein bound internal standards were synthesized according to the procedure previously described by Butterworth et al. with modifications. 5 mg (0.018 mmol) of [^2^H_5_]-E_2_ (dissolved in 2 mL of acetone) were added to 3 mL of 10% acetic acid (v/v). After the addition of 50 mg of potassium nitrosodisulfonate, the mixture was shaken for 15 min at room temperature. A second portion of potassium nitrosodisulfonate (50 mg) was added and the reaction was continued for another 15 min. The quinones were extracted from the solution three times with chloroform (2 mL × 3). Chloroform was removed under a gentle stream of N_2_. Twenty μL of acetonitrile was added to the residue and reactions were carried out by incubating estrogen quinone with 100 mg human serum Alb at 37 °C for 2 h. The reactions were terminated by adding 10 mM of ascorbic acid (final concentration) and by chilling in an ice bath. The modified proteins were purified by dialysis against 4 × 4 L of 1 mM ascorbic acid at 4 °C for 24 h using Spectra-Por 2 dialysis tubing (MWCO 12,000–14,000). The dialyzed proteins were lyophilized, weighed, and stored under − 80 °C prior to use. The oxidation of [^2^H_5_]-E_2_ to its corresponding reactive quinones mediated by potassium nitrosodisulfonate provided a direct procedure of synthesizing deuterated analogs included [^2^H_4_]E_2_-2,3-Q-1-S-Alb, [^2^H_3_]E_2_-2,3-Q-4-S-Alb, and [^2^H_3_]E_2_-3,4-Q-2-S-Alb. A Fenton-type hydroxyl radical-generating system was employed to synthesize deuterium-labeled internal standards of naphthalene-derived quinones from [^2^H_8_]naphthalene^43,44^.

### Isolation of human serum albumin

Plasma samples collected from patients and controls were isolated from 5–10 mL of human whole blood after mild centrifugation at 1,000 × g for 10 min and kept at − 80 °C before use. Human serum Alb was isolated from plasma samples according to our previous method^15^. In brief, after the sample was returned to room temperature, a saturated ammonium sulfate solution was added dropwise thereto until the final concentration of ammonium sulfate reached 2.5 M (63% of saturation). The mixture was vortexed and immunoglobulins were removed by centrifugation at 3,000 × g for 30 min. Protein was purified by dialysis against 4 × 4 L of 1 mM ascorbic acid at 4 °C using Spectra-Por 2 dialysis tubing (MWCO 12,000–14,000). Dialyzed proteins were lyophilized, weighed, and stored at − 80 °C until analysis.

### Measurement of Alb adducts of estrogen quinones and naphthoquinones

All cysteinyl adducts arising from naphthoquinones and estrogen quinones were assayed as described by Waidyanatha et al.^44^ with minor modifications^34^. Briefly, isotopically-labeled protein-bound internal standards for naphthoquinones and estrogen quinones were added to a 8-mL vial containing 10 mg of protein. After drying in a vacuum oven, 750 μL trifluoroacetic anhydride was added and the reaction was allowed to proceed at 110 °C for 30 min. After cooling to room temperature, 20 μL methanesulfonic acid was added and the mixture was heated at 110 °C for an additional 30 min. After cooling to room temperature, un-reacted anhydride was removed under a gentle stream of nitrogen. Next, 1.5 mL of hexane was added to the residue. The hexane layer was washed twice with 2 mL of 0.1 M Tris buffer (pH 7.4) and once with 1 mL of deionized water. After concentrating the samples to 50 μL, 2-μL aliquots were analyzed by gas chromatography-mass spectrometry (GC–MS), Agilent 6890 series gas chromatography (GC) coupled with Agilent 5973 N mass spectrometry (MS). An HP-5 ms fused silica capillary column (length 30 m, inner diameter 0.25 mm, film thickness 0.25 μm) was used with 99.999% He as the carrier gas, and the flow rate was 1 mL/minute. The MS transfer-line temperature was 250 °C and the pressure of the chemical ionization reagent gas methane was 2.3 × 10^−4^ torr. In all cases, the ion source temperature and injection-port temperature were set at 150 °C and 250 °C, respectively. The GC oven temperature was held at 75 °C for 2 min then increased at 6 °C /minute to 145 °C, where it was held for 10 min. Late-eluting compounds were removed by increasing the oven temperature at 50 °C/min to 260 °C, where it was held for 5 min.

Quantitative analysis of adducts was performed using the above GC–MS method and the MS was set to the selected ion monitoring mode. By adopting our previously established protocol^35^, the fragment ions were monitored in the negative ion chemical ionization mode for their respective trifluoroacetic acid derivatives. A standard curve was drawn with a concentration range of 0.1–500 pmol.

### Precision and limit of detection of the assay

Based on a signal-to-noise ratio of 3, the limit of detection of the assay corresponds to 10 pmol/g for all adducts, assuming that 10 mg of protein was used. In some experiments, co-elution of samples and authentic standards was performed by spiking samples with E_2_-2,3-Q-1-S-NAC, E_2_-2,3-Q-4-S-NAC, E_2_-3,4-Q-2-S-NAC, 1,2-NPQ-Alb, and 1,4-NPQ-Alb (25 pmol) followed by analysis of the mixture according to the previous protocol^15^. The precision, as indicated by estimated coefficients of variation, was less than 20% for all adducts, including 1.4-NPQ-Alb, 1,2-NPQ-Alb, E_2_-2,3-Q-4-S-Alb, E_2_-3,4-Q-2-S-Alb (n = 3–5).

### Statistical analysis

The data are expressed as mean ± standard deviation (SD). All the data were log-transformed and tested for normal distribution by the Kolmogorov–Smirnov test. Linear correlations were investigated between individual adduct levels by simple regression. Linear discriminant analysis was conducted to distinguish individuals with or without breast cancer. IBM SPSS Statistics version 20 was used to perform statistical analyses.
